# Data Analysis of Dynamics in Protein Solutions Using Quasi-Elastic Neutron Scattering—Important Insights from Polarized Neutrons

**DOI:** 10.1021/jacs.4c06273

**Published:** 2024-10-03

**Authors:** Mona Sarter, J Ross Stewart, Gøran Jan Nilsen, Mark Devonport, Kirill Nemkovski

**Affiliations:** STFC Rutherford Appleton Laboratory, ISIS Neutron and Muon Facility, Chilton, Didcot OX11 0QX, U.K.

## Abstract

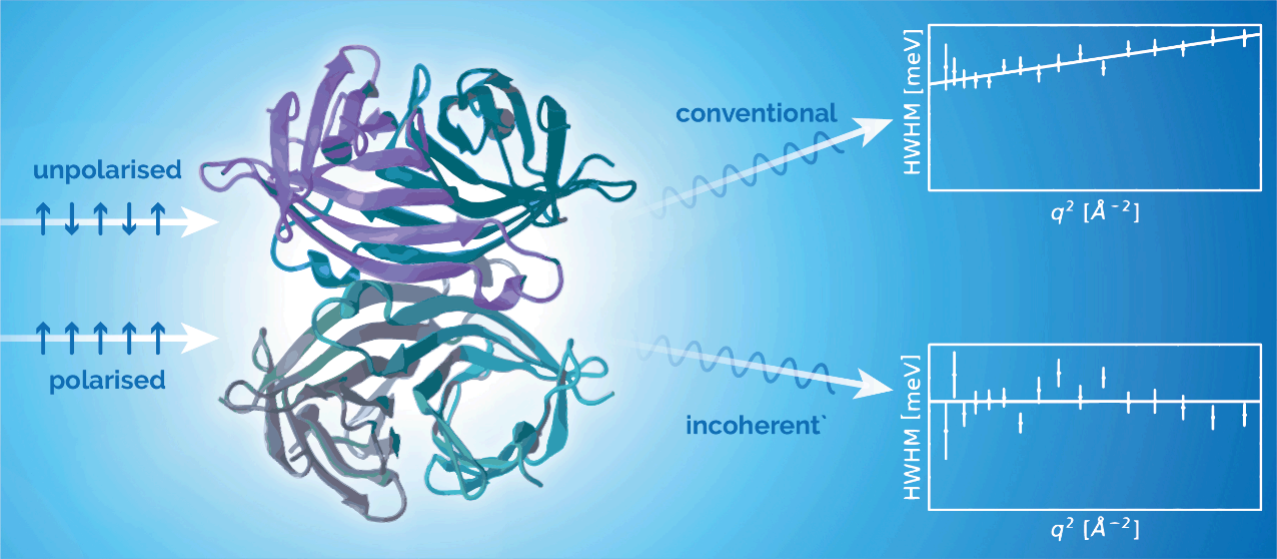

Protein dynamics play a vital role in biology. Quasi elastic neutron scattering (QENS) is an ideal method to access these dynamics. To isolate protein dynamics, it is important to separate the signal of the buffer and the protein. Normally data analysis is performed based on the assumption that the scattering spectrum is incoherent. To observe the full range of protein dynamics, it is necessary to perform the experiments in solution. This solution is usually a fully deuterated buffer, while the protein remains protonated. It is generally assumed that subtracting the buffer contribution removes all coherent signal from the measured spectrum, and the rest can be considered as purely incoherent. Up until recently, there was no way to experimentally verify this assumption. Polarized QENS experiments allow for the coherent and incoherent contributions to be separated. By comparing the results from the polarized QENS experiment and the standard analysis method from unpolarized QENS, we are thus able to check this assumption experimentally. We show that the pure incoherent spectrum obtained from polarization analysis does not match the results for unpolarized QENS. We discuss the implications of this for data analysis and possible solutions to the problem, as well as mitigation techniques for standard QENS.

## Introduction

Quasi-elastic neutron scattering (QENS) is one of the leading methods to obtain time- and space- resolved information about molecular dynamics in a wide range of systems relevant to biology and the life sciences. One way to enhance the quality of the information that can be extracted from QENS experiments is to use neutron polarization analysis (NPA), where polarized beams–beams in which the neutron spins are aligned–are used to separate the components of the neutron scattering cross section. In the case of nonmagnetic systems, this means separating the coherent scattering, which includes information about both collective and self-dynamics, from the incoherent, which probes only the latter. Improvements in neutron instrumentation and neutron sources have made wide-angle NPA a viable option for QENS instruments in recent years.^1−4^ However, many spectrometers with NPA were originally designed for a very different purpose and often are not suitable or, at least, not optimal for studying QENS.^3^ In particular, there is a lack of polarized instruments with both a high enough count rate and resolution as well as a wide scattering-angle coverage to simultaneously probe a broad range of length scales. Consequently, the number of NPA QENS experiments undertaken still remains very small. The normally accepted assumption for unpolarized QENS is that any potential coherent contribution is negligible and the observed scattering spectra can be treated as incoherent.^5−7^ This is based on the calculation of the total coherent and incoherent scattering cross sections for the samples by taking their isotopic compositions into account where the incoherent scattering from hydrogen dominates the total scattering cross section. Previous research using polarized neutron diffraction has shown that for small momentum-transfer (q) a non-negligible coherent contribution can be observed for protein in hydrated and solvated states and that for SANS and diffraction this needs to be considered for data analysis.^8^ Recent research using NPA QENS indicates that the dynamics of pure solvents, as well as the hydration layer of hydrated protein powders has a non-negligible coherent contribution.^9^ Furthermore, for hydrated protein powders there is a risk that a relevant component of the observed dynamics is caused by H/D exchange at the protein D_2_O interface.^10^ When working in solution this is no longer the dominant factor and thus unlikely to dominate the dynamics.^11^ However, this does not rule out other relevant coherent effects for proteins in solution. To the best of our knowledge the assumption - that the coherent contribution is negligible and that QENS data dominated by H scattering can be treated as incoherent - has never been checked, nor has any study been carried out to see how this affects QENS on proteins in solution specifically. In addition, to a discussion of the contribution of polarization to diffraction this was implicitly discussed in the past for conventional spectrometers such as spin–echo, but not QENS.^12^ This is largely due to NPA incurring a loss in count rate of around a factor of 10 compared to unpolarized neutrons (although some of that loss is compensated by an increase in signal-to-noise). Recent improvements in instrumentation provide the ability to experimentally verify or refute the discussed assumption. In this study we aim to answer if the inherent loss of flux in NPA QENS comes with a comparable qualitative increase in understanding of standard samples. At the conclusion of this paper we hope to have convinced the reader that the loss in flux is an acceptable, and often necessary, sacrifice in order to enable accurate quantitative analysis of the QENS signal.

Protein dynamics and conformational changes play an important role in biology.^13−15^ In order to observe the full range of a protein’s dynamics and conformations it is necessary to observe the protein in solution.^15−17^ Since QENS is sensitive to dynamical processes on molecular time- and length-scales, it is a particularly versatile and applicable technique for proteins in solutions, probing domain vibrations, side-chain fluctuations and conformational transitions, as well as diffusion at the molecular scale. It is also capable of differentiating between diffusion and confined dynamics. The time scales available are comparable to those of molecular dynamics (MD) simulations, making the technique particularly appropriate for the direct comparison of theoretical predictions with experimental results.^5,7,18^ The biological relevance of these systems highlights the importance of obtaining accurate results and verifying that all assumptions upon which the data analysis is based are correct. Conventional analysis of unpolarized QENS data assumes that the scattering is incoherent and that any potential coherent contribution can be neglected.^6,19,20^ One advantage of neutron scattering is that the method is isotope sensitive, which makes it ideally suited for biological experiments: Hydrogen ^1^H, which is abundantly present in proteins, has a large incoherent scattering cross section of σ_inc_ = 80.27 barn, while its coherent contribution is significantly smaller with σ_coh_ = 1.76 barn.^21^ Compared to this, deuterium ^2^H (D) has a larger coherent contribution σ_coh_ = 5.92 barn, with a small, but significant incoherent contribution σ_inc_ = 2.05 barn.^21^ By using deuterated buffers based on D_2_O instead of H_2_O, proteins can be observed in solution with the incoherent scattering from the protein being the dominant contribution. It is commonly assumed that by subtracting the volume-fraction weighted data measured on the buffer without protein, any non-negligible coherent contribution is removed from the data.^6,19,20^ For this to be the case, several assumptions are made; namely (i) that the bulk behavior of the D_2_O based buffer is comparable to the buffer interacting with the protein, or that the interaction of D_2_O and protein is negligible, (ii) that the hydration layer provides a negligible coherent contribution due to its being deuterated, and (iii) that any coherent contribution both from ^1^H, as well as other atoms in the protein, are negligible, or can be assumed to be accounted for by subtracting a background. Previous works highlight the problem of coherent contamination in incoherent neutron scattering.^8−10,22^ In particular, work on solvents and hydrated protein powders illustrates the need to investigate proteins in solution, as these systems may be especially sensitive due to the large fraction of sample that consists of D_2_O.^9,10^ For proteins in solution, concentrations can be as low as  in rare cases, while the upper limit of interest and in solution would be a living cell. For experiments focused on proteins, the highest concentration would be , representing self-crowding conditions.^7^ Even for this high concentration half the sample volume consists of D_2_O. Since the D_2_O concentration is typically higher, it is usually the dominant component of the sample.

In order to check experimentally the typical assumption that QENS data can be considered to be incoherent scattering with only a negligible coherent contribution, we performed QENS experiments on a protein in solution on the LET spectrometer at the ISIS Neutron and Muon Source (Didcot, UK) using NPA. These data allow for the separation of coherent and nuclear-spin-incoherent scattering, as well as the sum of both in order to provide a total scattering profile identical to that which would have been obtained in an unpolarized QENS measurement. The results obtained from these scattering spectra were then compared after identical data treatment. Streptavidin (STV) was chosen as a model protein for this experiment, since it is a symmetrical homo tetramer that does not have any significant structural flexibility or hinge bending motion, which could make data analysis more complex. Additionally, it has been comprehensively characterized so that no as-of-yet unexplained results are expected to occur for the different resolutions chosen, which might complicate the data analysis.^23,24^

## Materials and Methods

Sample Preparation. As described in previous publications, STV (13–139) was bought commercially (ProSpec-Tanoy TechnoGene Ltd., Israel, Rehovot, catalogue number pro-791-c) with a monomeric weight of 13271.6 Da and used in its stable tetrameric form. The protein was desalted using PD-10 desalting columns (Cytiva, UK) and incubated in D_2_O (99.9 atom D) for 36 h to exchange any freely exchangeable H atoms for D. The protein was kept dry after liophilization to avoid the risk of a humid environment triggering unwanted H/D re-exchange. This lyophilized powder was then reconstituted in a D_2_O-based buffer (TRIS-HCl, 120 mM NaCl, 5 mM KCl, 3 mM MgCl, 99.9% atom D, pH = 7.4) and the protein concentration determined as  using UV–vis (Gene-Quant 1300) with the absorption coefficient ε_STV_ = 167760 L mol^–1^cm^–1^.^23,24^

Neutron scattering experiments were performed on the direct geometry cold neutron multichopper spectrometer LET using NPA.^1,2,25^ The neutron beam is polarized using a m = 5 Fe/Si supermirror V-cavity and the scattered polarization is analyzed using a hyper-polarized ^3^He spin-filter cell which covers the entire solid angle (3 ster) of the detector array on LET. A current-ramped precession-coil π-flipper is placed after the polarizer enabling the measurement of non-spin-flip and spin-flip neutron cross sections as a function of neutron momentum transfer, q, and the neutron energy transfer, ℏω. The scattering function separation is carried out according to
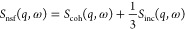
1
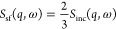
2Where *S*_inc_ is the spin incoherent dynamic structure factor and *S*_coh_ is the coherent dynamic structure factor, obtained separately by linear combinations of the measured non-spin-flip (nsf) and spin-flip (sf) cross sections corrected for the ratio of incident and scattered neutron momenta *k*_i_/*k*_f_.
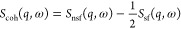
3
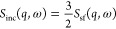
4where *S*_tot_ is the total dynamic structure factor.

Two different incident energies (E_i_) of 1.83 and 3.27 meV were simultaneously measured using repetition-rate-multiplication.^25^ These correspond to FWHM energy resolutions of 30.8 μeV, and 65.77 μeV respectively, with respective q-ranges of 0.35–1.55 Å^–1^ and 0.45–1.95 Å^–1^ and a q-integration step of Δq = 0.1 Å^–1^ for the total dynamic structure factor, as well as the separated coherent and incoherent dynamic structure factors. The NPA data was additionally reduced with a q-integration step of Δq = 0.15 Å^–1^. This was done to take the different relative statistics into account. This led to q-ranges of 0.525–1.575 Å^–1^ and 0.525–2.175 Å^–1^ for E_i_ = 1.83 and 3.27 meV respectively. All measurements were carried out at a temperature of 298 K. We measured the protein STV in solution, , as well as the separate buffer. Annular sample cans of 1 mm path length, which were filled to the top and sealed were used for all samples. Thus, the potential for H/D exchange with air was eliminated and it was ensured that the same amount of sample was in the beam for both measurements. Due to the buffer being a bulk solution the risk of H/D exchange during preparation is considered to be negligible.^11^ The ratio of transmissions is  In addition to these measurements, we measured at low temperatures (T = 2K where no QENS is expected) to determine experimentally the instrumental energy resolution for convolution during data analysis and an empty sample can measurement.

## Data Analysis

All collected data were converted to corrected incoherent, coherent and total dynamical structure factors *S*_inc_(q, ω), *S*_coh_(q, ω), and *S*_tot_(q, ω) using the standard LET python-based reduction script in Mantid, which accounts for the time-dependent polarizing efficiency of the instrument as well as path-length differences and neutron transmission through the ^3^He analyzer cell.^26,27^ Due to the total data being obtained by reassembling the spin flip and non-spin-flip data the difference in count rate between the total and incoherent data will not be several orders of magnitude, but instead around a factor of 2, depending on whether the coherent or incoherent scattering is dominant. The workflow for NPA corrections is described in depth in.^2^ The q-integration and energy-binning of the data used in the further analysis was then determined by plotting the full *S*(q, ω) data set in MSlice.^26,27^ This was done for the incoherent, coherent and total data. Each of these spectra contains contributions from the protein in solution, the buffer and the empty sample can. The empty sample can contribution was subtracted from all spectra. Afterward the buffer dynamic structure factor was subtracted from the protein in solution spectrum to isolate the protein scattering. This was done using the following:^28^
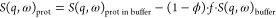
5Here ϕ =  is the volume fraction of the protein with the concentration  and the partial specific volume , and  is the factor correcting for neutron beam attenuation from protein and buffer.^23^

The total scattering function *S*_tot_(q, ω) that contains both incoherent *S*_inc_(q, ω) and coherent *S*_coh_(q, ω) is^6^
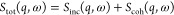
6

If the assumption that the coherent QENS contribution is negligible following buffer subtraction for proteins in solution holds true, it should be possible to analyze *S*_tot_(q, ω) and *S*_inc_(q, ω) using models appropriate for *S*_inc_(q, ω) (see eq 6, and all results obtained should be quantitatively identical within error. This means that any potential coherent contribution was deliberately not accounted for in the data analysis apart from a linear background being added, which amounts to the assumption that the coherent contribution is significantly faster than the incoherent. By doing so the assumption of no or only a negligible coherent contribution is tested. Therefore, the total and incoherent data were both analyzed using
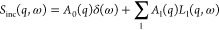
7Where *L*_l_(q, ω) is a Lorentzian function.

All confidence intervals on fit parameters are obtained from the diagonal elements of the covariance matrix of the fits. To decide between fits a Bayesian analysis was used in addition to knowledge of the physical dynamics of the sample. This is discussed in the Supporting Information.

## Results and Discussion

In order to compare the results for unpolarized QENS with incoherent QENS obtained from NPA, the data was reduced as described above. At first, the total spectra were analyzed, which is equivalent to the results from non NPA QENS. All analysis steps were performed for both resolutions. To improve readability the results will first be presented for the lower resolution of 65.77 μeV for the total and incoherent data, then the higher resolution 30.8 μeV results will be presented in the same way.

In order to be precise and consistent, the approach taken for the data analysis will be briefly laid out. Our aim is to use the simplest model that describes the data well and fits the spectra for the system, in this case STV, in order to avoid overfitting. This means that the simplest model will be tried first and if the fit is satisfactory, χ^2^ < 2, in relation to, in addition to the fit presenting a physically reasonable description of the data, we will consider the model to describe the data adequately. Due to the main information being in the peak the residues will be a necessary addition to χ^2^. If one or both of the above criteria are not met, a fit function that is one level up in regard to complexity (here, this corresponds to including an additional Lorentzian) will be tried. If a fit function of increasing complexity can be considered unphysical it will not be tried. This process will be continued until a satisfactory fit is achieved, at which point no more complex function should be tried as this would be unphysical. This approach was corroborated by Bayesian model selection, the details of which are presented in the Supporting Information.^27,29^

We will deviate from this approach here in order to compare the results for the total and incoherent data sets; if one data set requires a more complex function than the other, both data sets will be fitted with the same function. This is purely for a qualitative comparison and an illustration of the differences in data, as such a fit would and should not be attempted in the normal course of analysis. For the sake of comparison, this approach will be adopted throughout the manuscript.

Therefore, the first (and simplest) model tested was:
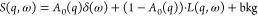
8Here *A*_0_(q) is the elastic incoherent structure factor (EISF), a linear background bkg is fitted, and the Lorentzian *L*(q, ω) is described by
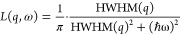
9

**Figure 1:**
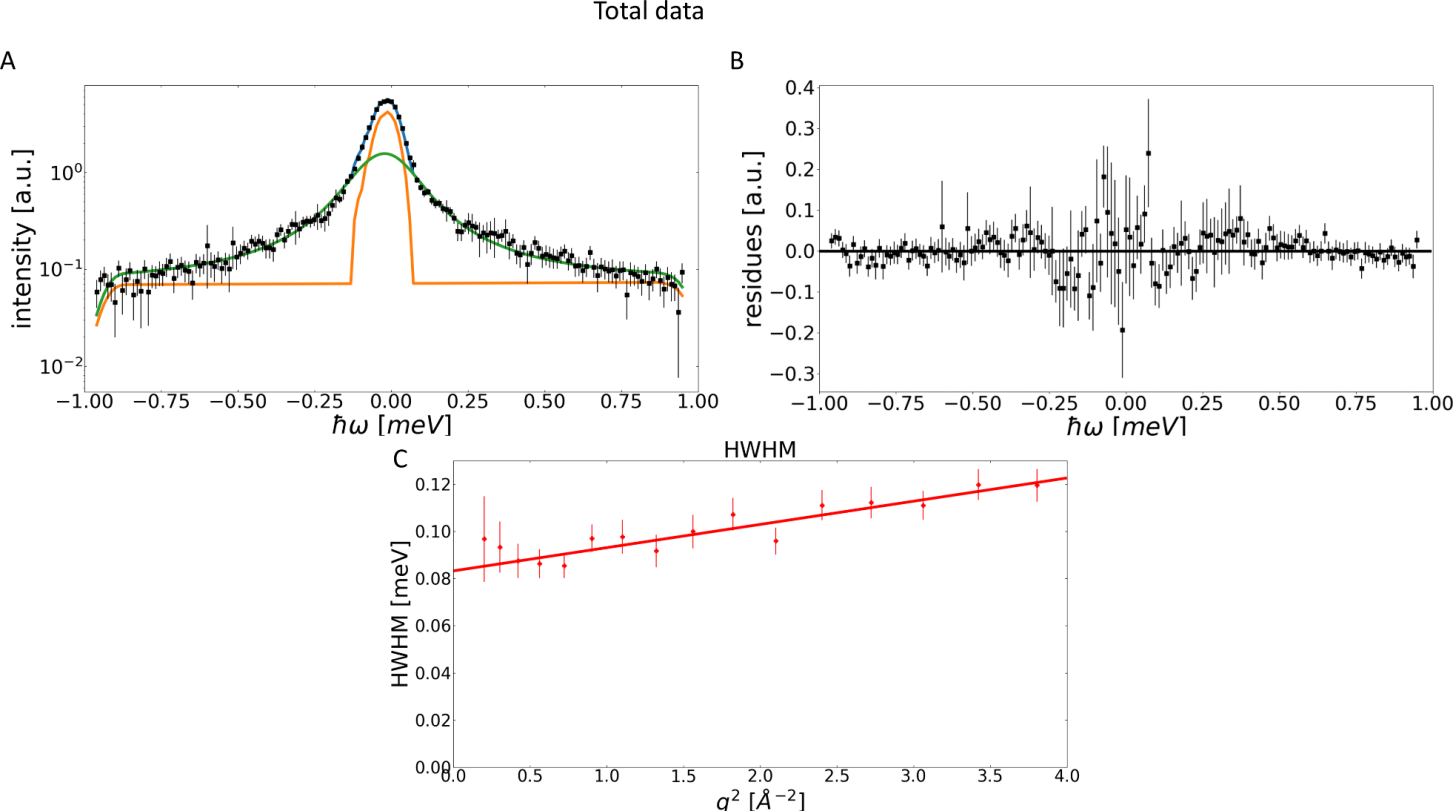
Illustration of the problems encountered when treating total QENS data over the whole q-range as incoherent. Panel (A) shows an example of the fit using eq 8 to *S*_tot_(q, ω) at q = 1.45 Å^–1^, for the resolution 65.77μeV. The *y*-axis is logarithmic, the total fit function is shown in blue, the data as black dots, the Lorentzian in green and the elastic contribution in orange. All are convoluted with the instrumental resolution function. Panel (B) shows the difference between the data and fit in panel (A). Panel (C) shows the HWHM peak width for *S*_tot_(q, ω) obtained from these fits. Here, it is apparent that a linear relationship to q^2^ is observed, making eq 8 not physically viable in this case.

All data were fitted for a q-range of 0.45 Å^–1^ – 1.95 Å^–1^, which is equivalent to the full q-range for the selected incident energy and resolution. An example of the fit function and its terms convoluted with the instrument resolution and fitted to the total experimental data is shown in Figure 1A, with the associated residual (data minus fit) in Figure 1B for *S*_tot_(q, ω). We observe that the fit curve lies slightly below the data but does fit the data reasonably well. However, to judge the quality of the fit it is also important to confirm that the fitted parameters are physically meaningful. By plotting the peak broadening HWHM against q^2^, a linear increase can be observed, see Figure 1C. For proteins in solution, this is indicative of global diffusive behavior, which could be observed on a time scale of 10–50 ps. However, for STV it is well documented that on this time scale internal motions should also be visible, which are not described by linear diffusion, but expected to be described either by localized confined motions or jump diffusive behavior  with Γ(0) = 0, as is appropriate for residues in a globular protein.^30^ Both of these scenarios would not yield a linear q^2^-ndependent behavior. This indicates that the fits using eq 8 are not physical and cannot be used to describe this data set.^24^

Therefore, this model is not considered to describe the data adequately and instead eq 10 was used.
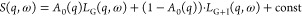
10
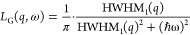
11
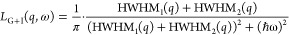
12

**Figure 2:**
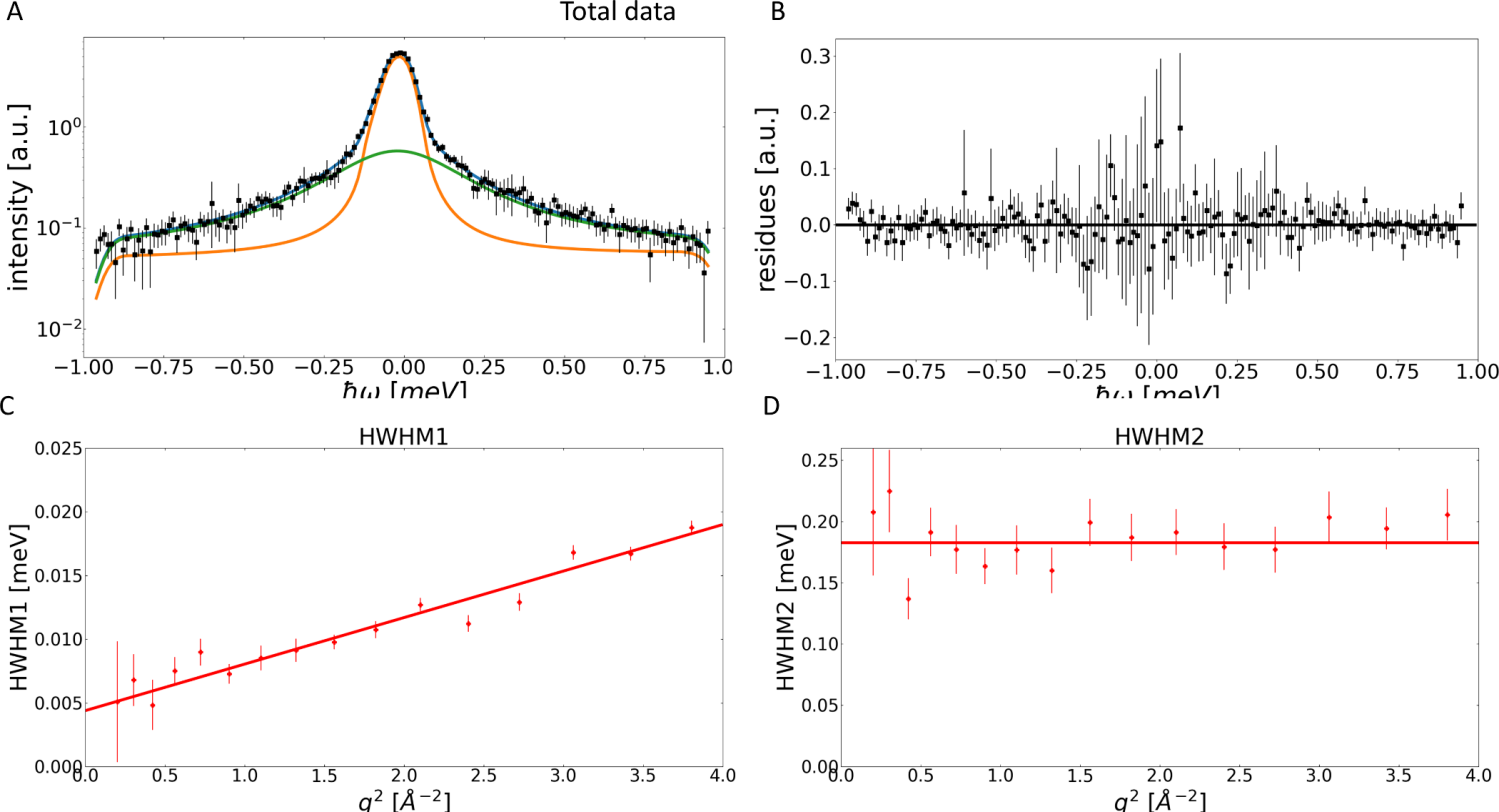
Panel (A) shows an example of the fit of eq 10 to *S*_tot_(q, ω) at *q* = 1.45 Å^–1^, for the resolution 65.77 μeV. The *y*-axis is logarithmic, the fit is shown in blue, the data as black dots, and the first Lorentzian in orange and the second Lorentzian in green. All are convoluted with the instrument resolution. Panel (B) shows the corresponding difference (data minus fit). Panels (C) and (D) show the peak width for the total data vs q^2^. Panel (C) shows that the first Lorentzian displays a peak width that shows a linear dependence with q^2^, which indicates diffusion. Panel (D) shows q^2^-independent behavior, which is indicative of local confined dynamics within the protein, *such as rotations or vibrations.*

This fit function assumes that 2 different dynamic contributions exist and uses one Lorentzian for each. The second Lorentzian eq 12 is the convolution of both dynamics experienced by the protein, the faster and the slower dynamic. Equation 10 is chosen if there is no seemingly immobile section to take into account due to the global and internal dynamics being both taken into consideration. Therefore, the amplitude before the δ− function becomes 0. As shown in Figure 2eq 10 fits the data very well. While the fit shown in Figure 1 based on eq 8 had a χ^2^ = 0.63, which already indicates an acceptable fit, this reduced to χ^2^ = 0.48 for Figure 2 based on eq 10. This indicates that the errors were overestimated, or systematically too high.

The peak width, as shown in Figure 2C, displays a linear relationship with q^2^, which describes the global effective diffusion of the protein in solution. This corresponds to an effective diffusion coefficient of , which is, within error, identical to the previously observed diffusion coefficient .^23^ This close agreement is surprising, as a reduced diffusion coefficient due to the observations taking place on the far faster 10–50 ps time scale as opposed to the ns time scale probed by the previous experiment was expected. In other words, the faster time scale of the present experiment should lead to an apparent reduction of the diffusion coefficient. Indeed, another recent publication found that at this time scale the diffusion coefficient could no longer be resolved at all.^24^ Despite this result appearing to be surprising and inconsistent at this stage, it is important to note that a reduced q-range compared to the current analysis was used in that work. The significance of this will become apparent during the course of the following discussion.

As previously discussed, the above results assume that for the purposes of the analysis, *S*_tot_(q, ω) can be treated as incoherent scattering. The buffer and empty sample holder subtraction to prepare the reduced data for fitting was described, as well as the assumption that after buffer subtraction any coherent contribution is negligible. In order to confirm this, all that is now required is to repeat the data analysis for *S*_inc_(q, ω) obtained from NPA. For this purpose, the same analysis steps described above for the total data are repeated. If the assumption that *S*_tot_(q, ω) is purely incoherent holds, this analysis should provide the same qualitative results and match the quantitative results within error. In addition to the Δq = 0.1 Å^–1^ integration increment the incoherent data was also analyzed with Δq = 0.15 Å^–1^ as an integration increment to take the reduced statistics into account. The wider integration is shown in the Supporting Information.

**Figure 3:**
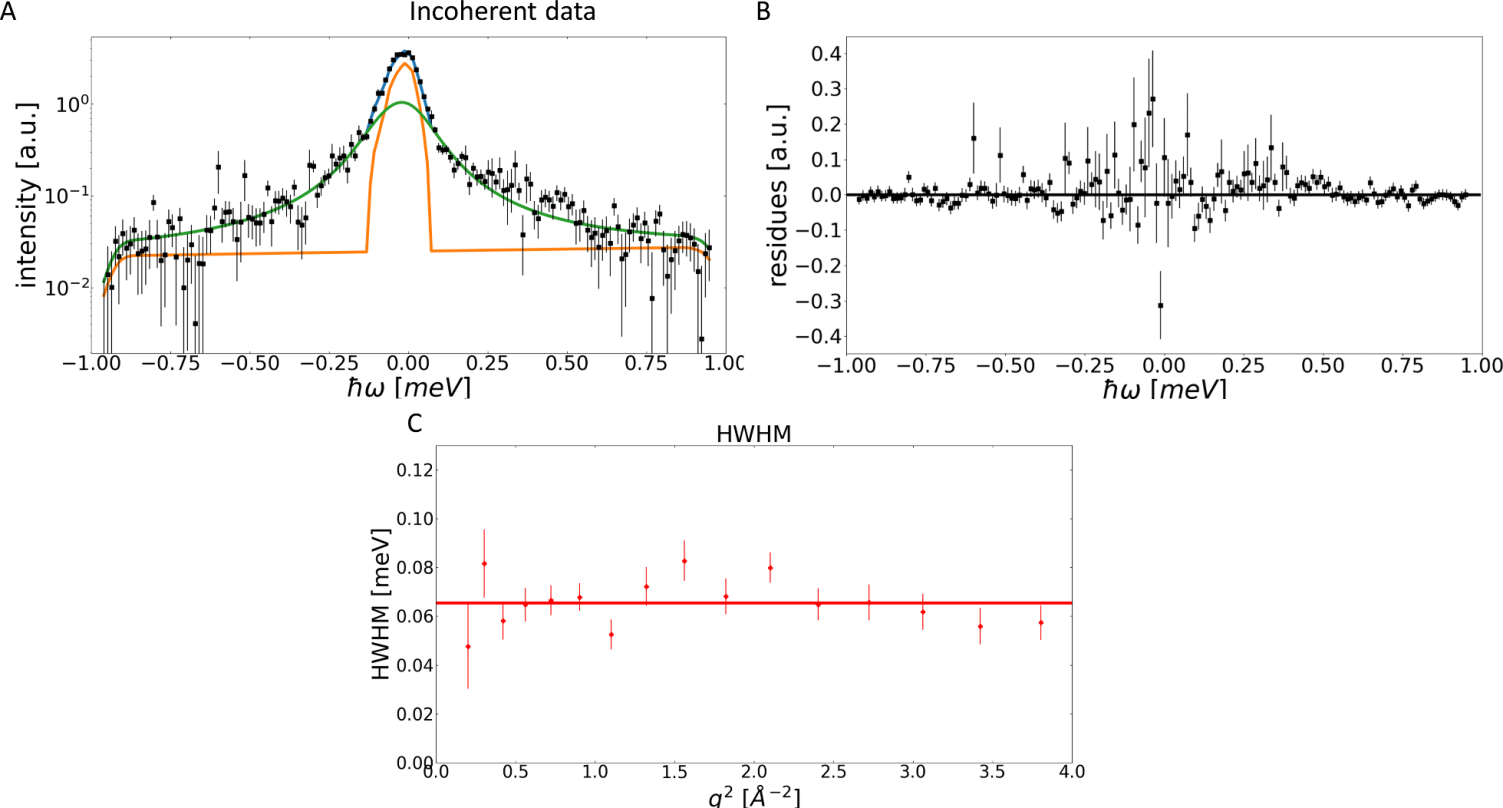
Panel (A) shows an example of a fit of eq 8 to *S*_inc_(q, ω)at q = 1.45 Å^–1^, for the resolution 65.77 μeV. The *y*-axis is logarithmic, the fit is shown in blue, the data as black dots, the Lorentzian in green and the elastic contribution in orange. All are convoluted with the instrument resolution. Panel (B) shows the corresponding difference (data minus fit). Panel (C) shows the peak width. Here, it is apparent that no q^2^ – dependence is observed.

All incoherent data obtained from NPA was treated the same way as the total data with the only difference being that the data sets for sample, buffer, and empty sample holder were the purely incoherent data. Thus, the data was first fitted with eq 8, as shown in Figure 3A. Unlike Figures 1C and 2C, Figure 3C does not show any indication of q^2^– dependence. Combined with the fact that the fit describes the spectra well, this is the point where data analysis would normally be considered finished, since the simplest physical model that describes the data well is nearly always the one that should be used If a smaller value of χ^2^ were found for a more complex fit function this improvement would most likely be caused by overfitting. Here χ^2^ = 1.25, which is indicative of an acceptable fit.

However, in this case, the aim is to compare unpolarized with NPA QENS and therefore for the sake of comparison eq 10 was used to fit the incoherent data as well. The results are shown and discussed in Table 1, as well as the Supporting Information.

**Table 1:** Diffusion Coefficients and Peak Broadening Obtained for the Different Approaches Obtained for Incoherent and Total QENS Data at 2 Different Resolutions 30.8 μeV and 65.77 μeV^a^

			
Resolution 65.77 μeV			
Total eq 8	15.0 ± 2.4	0.101 ± 0.012	–
Total eq 10	5.5 ± 0.4	–	0.181 ± 0.021
Incoherent eq 8	0 ± 3	0.064 ± 0.009	–
Incoherent eq 10	3.3 ± 0.6	–	0.144 ± 0.047
Incoherent 0.15 Å^–1^ integration eq 8	0 ± 3	0.066 ± 0.010	–
Incoherent 0.15 Å^–1^ integration eq 10	4.5 ± 0.4	–	0.150 ± 0.041
Resolution 30.8 μeV			
Total eq 8	6.6 ± 2.4	0.056 ± 0.010	–
Total eq 10	4.0 ± 0.4	–	0.137 ± 0.035
Incoherent eq 8	0 ± 3	0.038 ± 0.008	–
Incoherent eq 10	3.9 ± 0.5	–	0.145 ± 0.094
Incoherent 0.15 Å^–1^ integration eq 8	0 ± 3	0.040 ± 0.005	–
Incoherent 0.15 Å^–1^ integration eq 10	2.9 ± 0.4	–	0.148 ± 0.053

a Some of the diffusion coefficients amount to 0 indicationg that a model that does non assume a global diffusive behavior is appropriate for those datasets.

It is apparent that *S*_inc_(q, ω) is best described by eq 8, while *S*_tot_(q, ω) is best described by eq 10. Not only does this difference in fit functions result in quantitative differences it also assumes different underlying dynamics being observed, with eq 8 focusing on the protein’s internal dynamics, while eq 10 describes both the internal dynamics and the global diffusion of the protein.^6,20^ This is highlighted by the unphysical diffusion coefficient found for the incoherent data Table 1. The diffusion coefficient effectively amounts to 0. Indeed, no diffusion is observed and instead  is the value that should be used to interpret the proteins internal dynamics. This proves that the assumption that the coherent contribution to the scattering is negligible is wrong in this case. *S*_tot_(q, ω) differs from the incoherent and this is plainly due to a non-negligible coherent contribution.

Since the statistics for *S*_inc_(q, ω) are worse than for *S*_tot_(q, ω) the analysis was repeated using a wider q-integration increment, as described above. The results are displayed in Table 1, as well as Figures shown in the Supporting Information. The resulting fit parameters are consistent for both q-integration settings, indicating that the differences between the fits to the summed versus incoherent data are not due to the poorer statistics of the latter.

We now turn to the second resolution setting of 30.8 μeV. By repeating the analysis process for this setting we will clarify whether the observed discrepancy between *S*_tot_(q, ω) and *S*_inc_(q, ω) is resolution independent.

The same steps of fitting the total and incoherent data with eqs 8 and 10 each were repeated. Fitting eq 8 to *S*_tot_(q, ω) again provided the nonphysical result of a linear q^2^-dependence. While the extracted diffusion coefficient, D_eff_ = 4.0 ± 0.4 is smaller than the previously measured diffusion coefficient, this is not unexpected due to the faster time scale of 50–100 ps compared to ns. What is surprising, however, is that D_eff,30.8 μeV_ is smaller than D_eff,65.77 μeV_. The expectation would be for this to be the other way around, due to the slower time scales showing more of the dynamics previously observed. The expected diffusion coefficient of D_0, DLS_ = 6.03 Å^2^ns^–1^ means that the higher the resolution the more likely it is that it can be completely resolved.^23^ In addition, the higher resolution at 30.8 μeV compared to 65.77 μeV resolution had a considerably smaller q-range, the significance of which will become apparent later.

The fits and a more in-depth discussion of technical details can be found in the Supporting Information for *S*_tot_(q, ω)and *S*_inc_(q, ω) respectively.

For *S*_inc_(q, ω) the analysis was performed for Δq = 0.1 Å^–1^ and Δq = 0.15 Å^–1^ as q-integration intervals. In both cases eq 8 fits the data and provides a physical result with an acceptable χ^2^ and the results are identical for both Δq within error. For consistency the fit was repeated with eq 10.

These results mirror the previous results for the first resolution 65.77 μeV and confirm that the assumptions made for standard QENS do not apply to the second resolution of 30.8 μeV either. This indicates that the influence of the coherent scattering on the total QENS spectra is resolution independent, in this case.

This leads to the conclusion that, in STV, *S*_tot_(q, ω), which is what is measured in an unpolarized QENS experiment, and *S*_inc_(q, ω) obtained from NPA QENS cannot be adequately described using the same model. Instead, the separated incoherent spectra are described by eq 8 and the unpolarized QENS spectra are described by eq 10.

With this consistent deviation between the data collected by the different approaches now confirmed, an explanation is required. The obvious difference between the total and incoherent data sets is the presence of coherent contributions in the former. Therefore, the *S*(q) = ∫ *S*(q, ω)dω for the coherent, incoherent and total data, integrated over an energy range of 250 μeV and 80 μeV centered on the elastic line respectively will now be compared.

**Figure 4:**
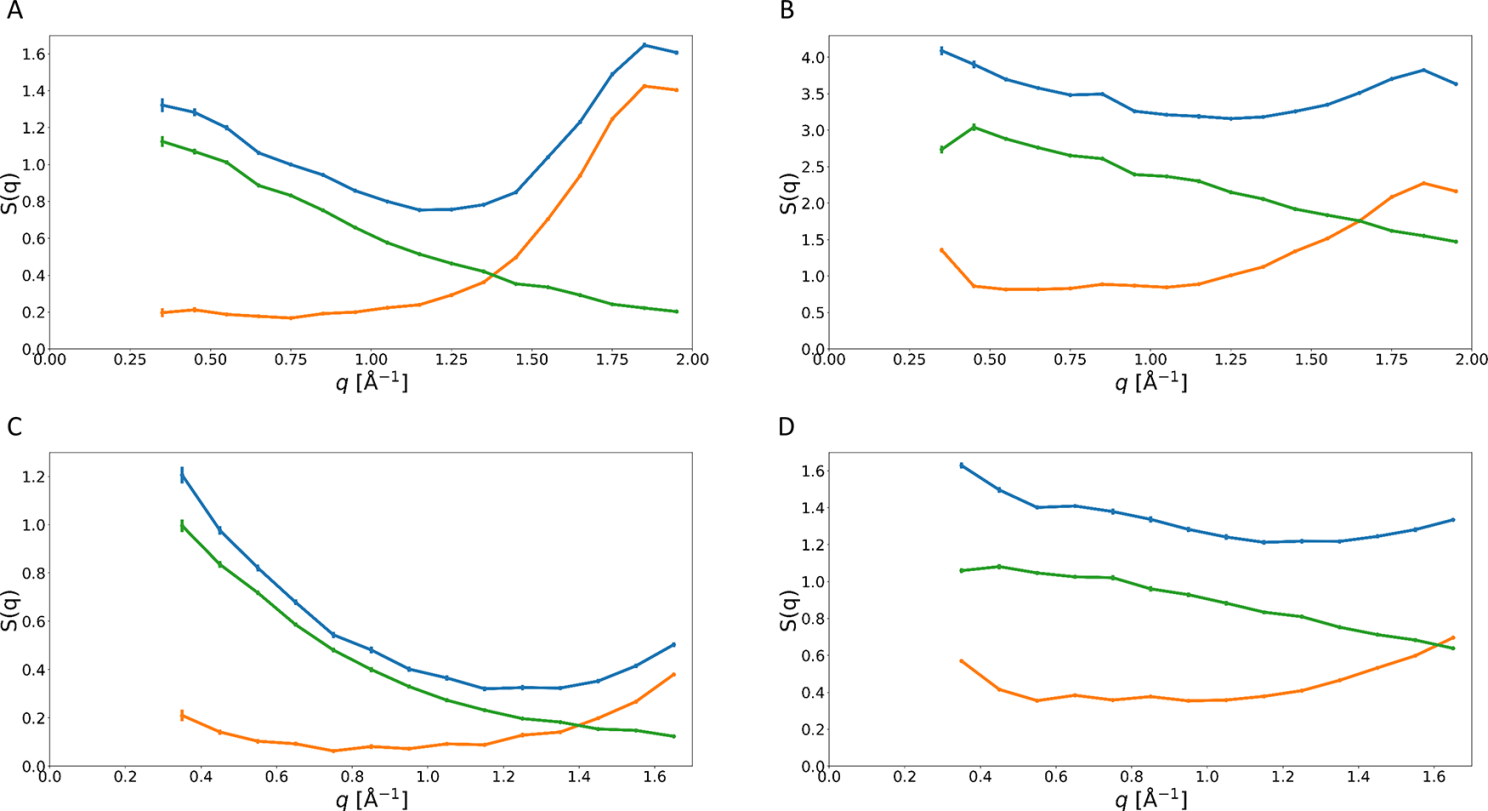
*S*_coh_(q) is shown in orange, *S*_tot_(q) in blue and *S*_inc_(q) in green. Panel (A) shows the *S*(q) components for the pure buffer at a resolution of 65.77 μeV. An increase in the coherent contribution can be observed, even though the buffer has been subtracted, which according to the assumptions should take care of the non-negligible coherent contributions. Panel (B) shows the *S*(q) components for STV at a resolution of 65.77 μeV. Here, it can be observed that for higher q, *S*_coh_(q) not only exceeds *S*_inc_(q), it also dominates the behavior of *S*_tot_(q). This matches previously observed behavior.^9^ Panel (C) shows the *S*(q) components for the buffer at a resolution of 30.8 μeV; here, it can be observed that for higher q, *S*_coh_(q) not only exceeds *S*_inc_(q), it is the dominant contribution to *S*_tot_(q). Panel (D) shows *S*(q) components for STV at a resolution of 30.8 μeV. An increase in the *S*_coh_(q) can be observed, even though the buffer has been subtracted.

*S*(q) is displayed in Figure 4A,B for the 65.77 μeV resolution. It is immediately apparent that for higher q the coherent contribution for STV Figure 4B, as well as the buffer Figure 4A is no longer negligible. Instead, the coherent scattering for the former has a higher intensity than the incoherent contribution and shapes the behavior of the total *S*(q). For the buffer this is even more pronounced. This highlights the need for highly accurate buffer subtraction. In addition, it shows that for higher q, unpolarized QENS spectra of proteins in solution should not be treated as purely incoherent, and instead a non-negligible coherent contribution remains present. In addition to a remnant contribution from the buffer this could also be the coherent contribution from the protein itself, since ^1^H has a nonzero coherent scattering contribution.

The higher resolution data for ΔΕ = 30.8 μeV is shown in Figure 4C,D and the effect described above can again be observed. However, it is not quite as pronounced. This is due to the limited q-range. When comparing the results for similar q-ranges, it can be seen that the observations are identical for both resolutions.

These *S*(q) confirm what we previously found from comparing the fitting of the quasi elastic spectra for both resolutions. Irrespective of the resolution the total data shows a non-negligible coherent contribution even after buffer subtraction. In addition, *S*(q) shows the same behavior previously observed by Gaspar et al.^8^ in their paper discussing NPA for SANS-WANS (small angle neutron-scattering to wide angle neutron-scattering) experiments. This suggests that the results we observe for STV as a model system might apply to other proteins more generally.

The most probable cause for the remaining coherent contribution is the hydration layer surrounding the protein. It is not subtracted during buffer subtraction due to the difference between bulk and hydration water behavior, and the coherent contribution of ^2^H in the hydration layer is larger than that of ^1^H in the protein. This means that a non-negligible coherent contribution of unknown size needs to be taken into account for any non NPA QENS experiment. Even taking the other atoms in the protein into account the total coherent scattering cross sections are σ_coherent, streptavidin_ ≈ 7300 barn and σ_coherent, hydrationlayer_ ≈ 61600 barn based on literature values for the amount of water molecules.^31^ The scattering cross sections are statistical values, which can be used as a first indicator to determine the sample component most likely to contribute to *S*_inc/coh_(q).^10,32^ One of the limitations of σ is that the q-dependence of the coherent and incoherent contribution is not reflected in the total value. This means that information from *S*_inc/coh_(q) obtained from NPA experiments is vastly superior, again emphasizing the need for additional instruments with this capability.

We will now discuss how to analyze the data to avoid coherent contamination. The suggestions will range from performing the whole experiments using NPA QENS to what to do if no NPA is available at all.

While the obvious solution to this problem would be to only perform QENS experiments with NPA from now on, the availability of NPA QENS is extremely limited. It is therefore necessary to consider how to utilize this information to avoid the coherent region for non NPA QENS in this interim period before the NPA QENS capability is built up.

While it is easy to suggest that avoiding the dominantly coherent region in q is the easiest solution, it is of course necessary to define carefully the methodology by which this region may be identified.

**Figure 5:**
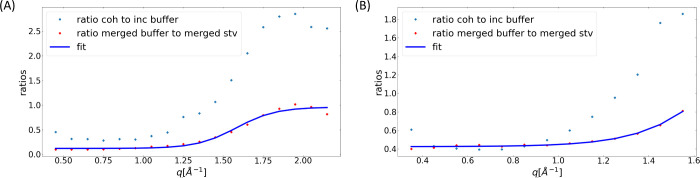
Panel (A) shows the ratio of coherent to incoherent integrated buffer signal, as well as the ratio of the integrated STV spectrum to the integrated buffer spectrum for the total data for the resolution 65.77 μeV. Panel (B) shows the same for resolution 30.8 μeV. In both cases, the turning point and ratio of coherent to incoherent of 1 position correspond to q = 1.15 Å^–1^.

If, as in this case, *S*(q) information from NPA is available, the q-space that is accessible without encountering the area where coherent contamination becomes significant can be determined easily. The maximum q-value could, we suggest, be determined by plotting the ratio of the coherent signal to the incoherent signal of the buffer Figure 5. Any q-range for which this ratio exceeds the value of 0.5 are summarily no longer considered for data analysis. While a ratio of 0.5 indicates that there is a 50% coherent contribution and this might be considered to be excessive, it is important to remember that after the buffer subtraction, only a very small amount of buffer (the hydration layer) continues to contribute to the signal. Therefore, this ratio seems acceptable since the aim is to obtain reliable data while keeping the largest q-range possible. We propose to use the same ratio irrespective of sample concentration. While for a very concentrated solution the ratio of bulk: surface D_2_O will decrease suggesting that the subtraction of the buffer introduces a larger error than it would for a lower concentration, this is compensated by the experimental uncertainty in the concentration itself. This systematic uncertainty is constant, thus decreasing in its influence on the final result as the concentration increases.

It is of interest to note that the analyzable maximum q is now limited to 1.15 Å^–1^ for 65.77 μeV and 0.95 Å^–1^ for 30.8 μeV. While these values are not identical they are close together and even for 1.15 Å^–1^ the results for 30.8 μeV seem acceptable. The HWHM results for the fits using eq 8 are shown in the Supporting Information. This indicates that for each sample the accessible q-range can be determined by one *S*(q) and might in the future allow for a repository of the most commonly used buffers to decide on the accessible q-range. Characterizing *S*_inc_(q), *S*_coh_(q), and *S*_tot_(q) for a sample does not necessarily require the use of NPA QENS. NPA diffraction, which is both faster and comparatively more widely available could also be used.^8^

Due to the limited availability of NPA spectrometers it seems prudent to also provide an experimental solution for those users who cannot access these instruments. While NPA, or determining the *S*(q) for coherent and incoherent contributions should always be the first choice there we suggest an alternative for situations where this is not available.

This alternative approach is achieved by integrating the buffer intensity and the sample intensity for each q-value and plotting the ratio of the integrated sample intensity to the buffer intensity against q. The point at which the slope of the sigmoid begins to increase seems to be appropriate, especially since the resulting q-value corresponds to the one obtained from the ratio of coherent to incoherent signal as described above, Figure 5.

In order to confirm that limiting the q-range in this way is a valid approach the above-described analysis for incoherent and total QENS data was repeated for a maximum of q = 1.15 Å^–1^ that was limited to a mainly coherent area. It can be observed that for the limited q-range the total and the incoherent data can be described by the same fit function (eq 8), the average peak broadening indicating the locally confined or restricted dynamics of STV, such as amino acid side chain rotations Table 2.^33^ Also,  is larger for the total data than the incoherent data. While 2 data sets are not sufficient to confirm a trend this could very well be due to the non-negligible coherent contribution, see Supporting Information.

**Table 2:** Results of Fitting the Total Data Limited to a Maximum of q = 1.15 Å^–1^ and the Incoherent Data for the Full q-Range with eq 8 Are Shown Here

		
resolution 65.77 μeV		
total q-limit –0.95 Å^–1^	12.5 ± 21.7	0.091 ± 0.005
total q-limit –1.15 Å^–1^	11.4 ± 12.2	0.091 ± 0.005
total q-limit –1.35 Å^–1^	18.2 ± 7.3	0.094 ± 0.007
incoherent	0 ± 3	0.064 ± 0.009
resolution 30.8 μeV		
total q-limit –0.75 Å^–1^	60.6 ± 21.1	0.051 ± 0.013
total q-limit –0.95 Å^–1^	47.7 ± 10.7	0.054 ± 0.013
total q-limit –1.15 Å^–1^	14.3 ± 5.4	0.055 ± 0.011
incoherent	0 ± 3	0.038 ± 0.008

Due to the good agreement of the fit to the data and the realistic behavior of the peak broadening an additional fit with eq 10 was not performed. The obtained diffusion coefficient highlights the absence of a linear q^2^-dependence, confirming that the previously observed diffusion was not the center of mass diffusion but instead an artifact caused by the q-dependent coherent contribution to the total data. As shown in Table 2 increasing the q-range causes the fits to tend slightly toward the need for eq 10 to be used, while reducing the q-range does not seem to increase the agreement between the total and incoherent data significantly, meaning that the middle cut off of 1.15 Å^–1^ and 0.95 Å^–1^ respectively for the different resolutions are the most suitable.

## Conclusions

We have discussed the assumptions that go into the analysis of a typical unpolarized QENS experiment on proteins in solution, such as the calculation of the total scattering cross sections with coherent and incoherent contributions. Having now tested these assumptions experimentally we have come to the following conclusions.

Regarding the buffer subtraction, the majority of the buffer’s signal is removed by subtraction. However, any hydration layer that does not follow bulk dynamics is not subtracted. The total volume fraction of the hydration layer is determined by protein concentration and can be estimated using eq 13 for globular proteins. Here N_A_ is the Avogadro constant, M_w, protein_ the protein molar mass, d_hydration_ the thickness of the hydration layer, r_protein_ the protein radius, and c_protein_ the concentration in solution.
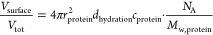
13

This leads to the second assumption that the coherent scattering contribution of the remaining buffer and the hydrogens in the protein itself is negligible. Regarding this we have found that this is not the case, revealed by the improved sensitivity of QENS instruments. This means that the assumption that automatically followed on from the first two; that after buffer subtraction and empty can subtraction and by including a linear background term in the fit, the data can be treated as incoherent, does not hold for all q-values. We find that this is the case only in the limit of low q-values, with the lowest q being required to be above the SAS range and that if higher q-values are included in the analysis the results between non NPA and NPA QENS data differ significantly.

Having concluded that the assumptions do not hold for the full q-range our results clearly highlight the need for NPA for all experiments that rely on their ability to separate the coherent and incoherent signal for data analysis, especially QENS. For proteins in solution it is recommended that if no NPA QENS is available, *S*_coh_(q) and *S*_inc_(q) should be measured to judge the appropriate q-range. This can be done using NPA diffraction instruments (such as D7 or D3 at the ILL), or a software capable of prediction *S*_inc_(q) and *S*_coh_(q) for a wide enough q-range based on the protein’s pdb structure.

If this is not possible the method using the integrated spectral intensity of protein and buffer described in Results and Discussion, Figure 5, should be utilized to ensure that the analysis is limited to the relevant q-range. In cases where polarized QENS is not available, we require the additional assumption that the coherent to incoherent ratio in *S*(q, ω) is similar to that estimated from *S*(q) for all ω. In addition, all absolute values determined have the potential to overestimate the peak broadening due to the non-negligible coherent contribution. However, a comparison between samples is still possible for conventional QENS and the coherent contamination of the absolute value can be taken into account by assuming an additional systematic uncertainty during data interpretation.

In the limited q-range the assumption that the signal is dominated by incoherent scattering and that the coherent contribution can be neglected for normal QENS model selection still holds. As instrumental sensitivity improves it is important to reverify this assumption, as the improved measurement accuracy might also allow for a greater influence of the weaker coherent scattering.

For simulations supporting the QENS data analysis it is important to take any H/D exchange both in the protein, as well as potentially in the buffer, into account. While simulated coherent spectra are useful and can be compared directly with the coherent spectra from NPA QENS in the data analysis, this provides several additional challenges for unpolarized QENS. Comparison of the simulated total (coherent) data to an incoherent spectrum is impossible and any mismatch between simulation and experiment might not be spotted. Another challenge is that not all proteins come with a known pdb structure, or indeed, even if a starting structure is known, the structure under the experimental conditions or with added ligands might be unknown thus preventing the use of simulations.

For a known structure the theoretical *S*_inc_(q) and *S*_coh_(q) could be calculated and used to determine a suitable q-range for unpolarized QENS.^10^

For future work it will be necessary to check if, as observed here, the coherent contribution is almost resolution independent. If that should be confirmed and in addition, the coherent contamination of identical buffers be protein independent, a database containing information on the accessible q-range for each standard buffer might prove to be the solution for unpolarized QENS until a sufficient amount of instruments is capable of NPA. Whatever the solution turns out to be, if a QENS experiment is carried out without NPA the most important step will be to investigate carefully what q-range can be considered reliable and to bear in mind during data analysis and interpretation that even in the best case scenario a contribution from the coherent scattering will be present. However, if the q-range is adequately limited there is no reason to disregard unpolarized QENS data. The wide range of resolutions available on the different instruments is of great importance for the full analysis of samples and the higher flux of unpolarized QENS a necessity for many experiments. Nevertheless, the long-term aim should be to provide NPA as a standard option on all QENS instruments.

## Data Availability

All data is freely available on DOI: 10.5286/ISIS.E.RB2220749.

## Supporting Information Available

Additional plots, more details on the data analysis performed, and discussing the Bayesian analysis and displaying the results (PDF)

## Abbreviations

QENS: quasi-elastic neutron scattering
NPA: neutron polarization analysis
SANS: small-angle neutron scattering
MD: molecular dynamic
FWHM: full-width half-maximum
EISF: elastic incoherent structure factor
HWHM: half-width half-maximum
WANS: wide-angle neutron scattering.
