# Supplementary material for: Data Analysis of Dynamics in Protein Solutions Using Quasi-Elastic Neutron Scattering—Important Insights from Polarized Neutrons
Source: J Am Chem Soc. 2024 Oct 3;146(41):28023–33. doi: 10.1021/jacs.4c06273 (PMC11488478; doi:10.1021/jacs.4c06273)
Supplement: Supplementary file 1 — ja4c06273_si_001.pdf [file ja4c06273_si_001.pdf]

Data analysis of dynamics in protein solutions using quasi-elastic neutron scattering – Important insights from polarized neutrons

Mona Sarter*, J Ross Stewart, Gøran Jan Nilsen, Mark Devonport, Kirill Nemkovski

STFC Rutherford Appleton Laboratory, ISIS 720 Neutron and Muon Facility, Didcot OX11 0QX, U.K

In this section we will discuss the data analysis that was left out of the main manuscript to avoid repetition for the reader who is interested in a more detailed discussion. In addition we will provide a graphical illustration of the influence of restricting the q-range for the total data. While also showing how a Bayesian analysis can be used to back up the decision whether Equation 8 or Equation 10 is more appropriate for a dataset.

Lower resolution data analysis $65.77\ \mu\mathrm{eV}$

All data was analysed as described in the 'Data Analysis' section. Following on from the fits to the total and incoherent data shown in 'Results and Discussion' here the fit of the incoherent data with Equation 10 will be shown and described, bearing in mind that this is only done for comparison to the total data, as such a fit is unphysical and would never be attempted during the normal course of data analysis. In additon the analysis will be repeated for the rougher $\Delta q = 0.15\ \text{Å}^{-1}$ integration for the incoherent data.

As can be seen in Figure S 1 A, Equation 10 provides a fit that describes the spectrum well and $\chi^2 = 1.09$. The fit was only performed here to compare the fit required for the total QENS data. However, it is important to note that Equation 8 already described the spectrum well. A diffusion coefficient can be calculated from the slope Figure S 1, however it is important to remember that the fit function assumed a q^2 −dependence and that in this case we are most likely looking at a case of overfitting that would not under normal circumstances have been seriously considered. Even though the fit was performed with identical parameters to the added data the resulting diffusion coefficient, Table 1, was smaller, emphasising that the data does not display the same behaviour. The fact that a diffusion coefficient could be obtained at all also highlights the dangers of overfitting by using an unphysical model. This model might then provide results that do not appear to be wrong. From this, the importance of providing an evidence based approach for data treatment regarding the coherent and incoherent contributions to QENS can easily be seen.

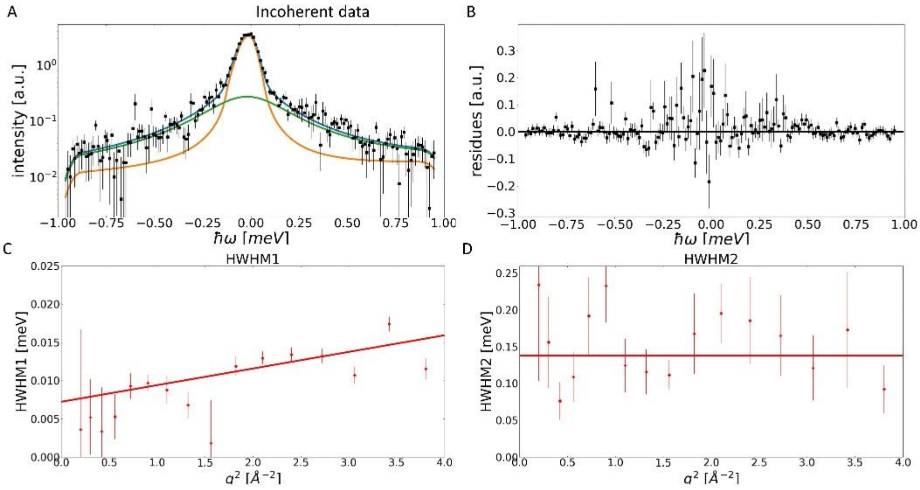

Figure S 1 A shows an example of the fit using Equation 10 for an incoherent spectrum at $q = 1.45\ \text{Å}^{-1}$, for the resolution 65.77µeV. The y-axis is logarithmic; the total fit function is shown in blue, the data as black dots, the first Lorentzian in orange and the second Lorentzian in green. All are convoluted with the instrument resolution. B shows the corresponding residues. C and D figure show the peak broadening for the incoherent. C shows that the first Lorentzian provides a peak broadening that shows a negligible linear dependence to q^2. D shows no q^2 - dependence for the second Lorentzian, which is indicative of localised constrained and restricted dynamics within the protein, and identical to what we saw for HWHM from Equation 8.

As discussed in the main manuscript 'Results and Discussion' the incoherent data has a lower count rate compared to the total data. In order to confirm that this does not influence the results the data analysis for the inconerent data was repeated using a rougher q-integration of $\Delta q = 0.15\ \text{Å}^{-1}$ to compensate for the reduced statistics. The resulting fits are shown here. For the resolution $65.77\ \mu\text{eV}$ fitted using Equation 8 see Figure S 2. This fit resulted in $\chi^2 = 1.30$, which indicates along with the residue plot shown that the data is described well by Equation 8. As before C indicates that no q^2-dependence can be observed, thus indicating that a shift to a more complex fit function is not necessary. However, as before due to the results observed for the added data the fits were repeated using Equation 10.

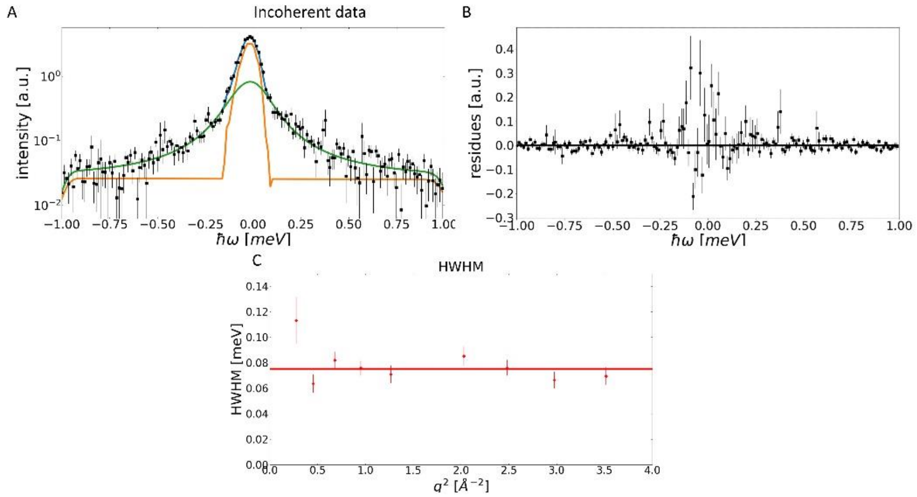

Figure S 2 The incoherent data at $65.77\ \mu eV$ resolution was analysed again using the rougher $0.15\ \text{Å}^{-1}$ q- integration. A shows an example of the fit using Equation 8 for the incoherent spectrum at $q = 1.425\ \text{Å}^{-1}$. The y-axis is logarithmic; the total fit function is shown in blue, the data as black dots, the Lorentzian in green and the elastic contribution in orange. All are convoluted with the instrument resolution. B shows the corresponding residues. C shows the peak broadening. It is apparent that no q^2- dependence exists.

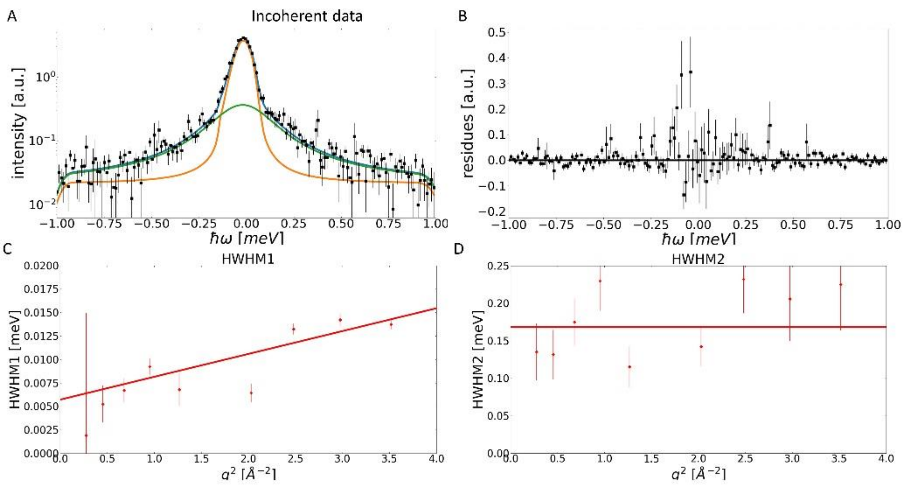

Figure S 3 The incoherent data at $65.77\ \mu eV$ resolution was analysed again using the rougher $0.15\ \text{Å}^{-1}$ q- integration. A shows an example of the fit using Equation 10 for the incoherent spectrum at $q = 1.425\ \text{Å}^{-1}$. The y-axis is logarithmic; the total fit function is shown in blue, the data as black dots, the Lorentzians in orange and green. All functions were convoluted with the instrument resolution. B shows the corresponding residues. C shows that the first Lorentzian's broadening provides a peak broadening that shows a linear dependence to q^2. D shows no q^2 −dependence for the second Lorentzian.

As can be seen in Figure S 3 Equation 10 provides a fit that describes the spectrum well and results in $\chi^2 = 1.15$. However, as before it is important to note that Equation 8 already described the spectrum well and an increase in the complexity of the fit function is only ever indicated if a simpler model does not adequately fit the data, or there is a specific reason why the simpler model cannot describe the data. As before a diffusion coefficient could be calculated (Table 1), but is the result of overfitting. Therefore, it is apparent that the incoherent data is best described by Equation 8, while the total data is best described by Equation 10. Not only does this difference in fit functions result in quantitative differences it also assumes different underlying dynamics, with Equation 8 focussing on the proteins internal dynamics, while Equation 10 describes both the internal dynamics and the global diffusion of the protein.

Higher resolution data analysis

As discussed in the main document 'Results and Discussion' the data analysis was repeated for the higher resolution data. The detailed steps and corresponding plots are shown here. The analysis was repeated for the second measured resolution $30.8\ \mu eV$. As before, the total and incoherent data were analysed separately, and again the total data, which corresponds to the results of normal non-polarised QENS was analysed first. The resulting fit to the spectra and corresponding residues can be observed in Figure S 4 A, B, where it can be seen that the curve obtained using Equation 8 already fits the data reasonably well and that $\chi^2 = 0.88$ also indicates a mathematically good fit. To confirm this, it is necessary to plot HWHM vs q^2, as can be observed in Figure S 4 C. Here it becomes apparent that the q^2- dependence is linear and therefore most likely describes the protein's diffusion in solution. This indicates that Equation 8 does not provide a physical model of the data.

On account of this observation, the spectra are therefore now fitted with Equation 10, which takes the global diffusion and the internal dynamics into account. These fits describe the spectra well $\chi^2 = 0.51$ Figure S 5 and in addition the behaviour of HWHM1 and HWHM2 corresponds to expectations, with a linear q^2-dependence for HWHM1 and no q^2-dependence for HWHM2.

This confirms the previously found results that the total data is best described by Equation 10.

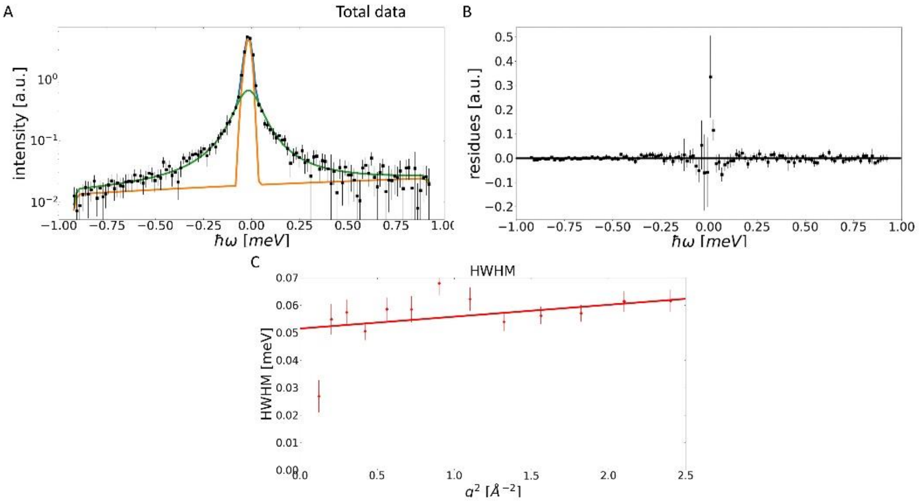

Figure S 4 A shows an example of the fit obtained using Equation 8 for the total spectrum at $q = 0.75\,\text{Å}^{-1}$ for the resolution $30.8\ \mu eV$. The y-axis is logarithmic, the total fit function is shown in blue, the data as black dots, the Lorentzian in green and the elastic contribution in orange. All are convoluted with the instrument resolution. B displays the corresponding. C shows the peak broadening for the added data plotted vs q^2. Here it becomes apparent that a potential q^2-dependence is observed.

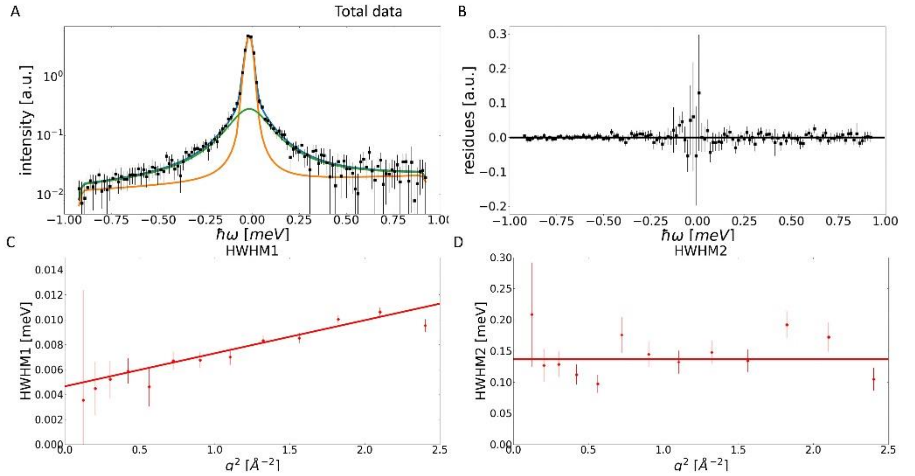

Figure S 5 A shows an example of the fit using Equation 10 for a total spectrum at $q = 0.75\ \text{Å}^{-1}$, for the resolution $30.8\mu eV$. The y-axis is logarithmic, the total fit function is shown in blue, the data as black dots, the first Lorentzian in orange and the second Lorentzian in green. All are convoluted with the instrument resolution. B shows the corresponding residues. C, D show the peak broadening for the added data. C shows that the first Lorentzian displays a peak broadening that shows a linear dependence with q^2, which indicates diffusion. D shows no q^2 dependence, which is indicative of localised constrained and restricted dynamics within the protein.

In addition the analysis was now repeated for the incoherent data.

To keep the results consistent and comparable the analysis was again started using Equation 8, which resulted in Figure S 6. The fits provided fit the data well $\chi^2 = 1.35$ and the HWHM in Figure S 6 C show no q^2 – dependency.

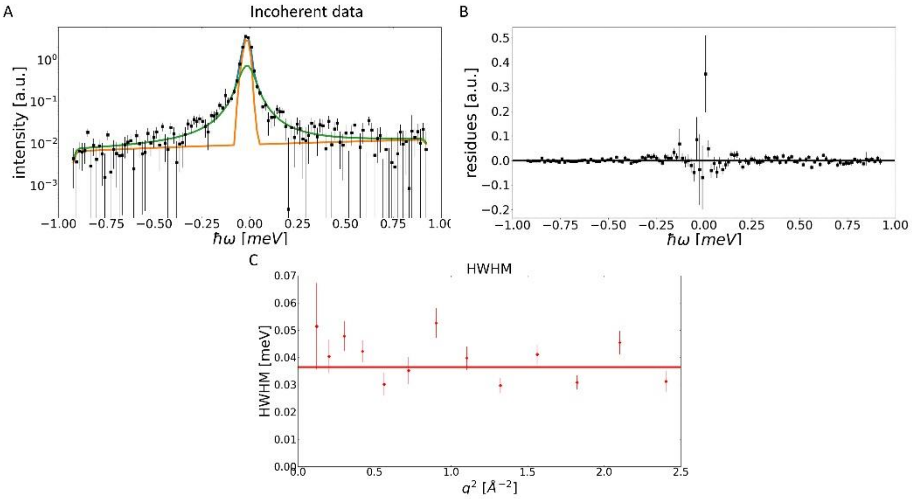

Figure S 6 A shows an example of the fit obtained using Equation 8 for an incoherent spectrum at $q = 0.75\ \text{Å}^{-1}$ for the resolution $30.8\ \mu eV$. The y-axis is logarithmic, the total fit function is shown in blue, the data as black dots, the Lorentzian in green and the elastic contribution in orange. All are convoluted with the instrument resolution. B shows the corresponding residues. C shows the peak broadening for the incoherent data obtained using by plotting the spectra using Equation 8. Here it becomes apparent that no q^2-dependence is observed.

In order to compare to the fits obtained from the total data as before the spectra were also fitted using Equation 10, as shown in Figure S 7. The fit fits the data well $\chi^2 = 1.14$. The peak broadenings of the fitted Lorentzians are displayed in Figure S 7 C. The diffusion coefficient obtained from the slope of HWHM1 in Figure S 7 C is smaller than that found for the total data. The fact that it is visible, as well as that a q^2 – dependence can be observed is due to this behaviour being given as expected in the fit. However, if a q^2 – dependence were present this would have already been visible in the peak broadening obtained from Equation 8.

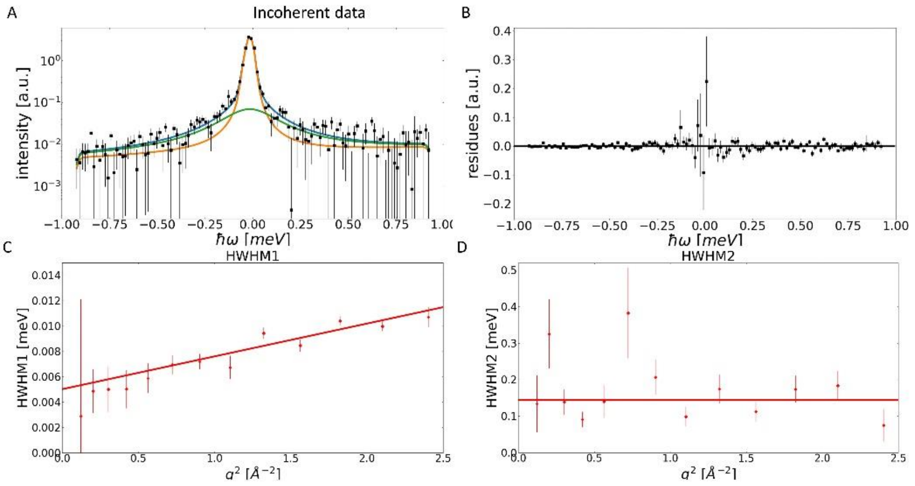

Figure S 7 A shows an example of the fit using Equation 10 for an incoherent spectrum at $q = 0.75\ \text{Å}^{-1}$, for the resolution $30.8\ \mu eV$. The y-axis is logarithmic, the total fit function is shown in blue, the data as black dots, the Lorentzian in green and the elastic contribution in orange. All are convoluted with the instrument resolution. B shows the corresponding residues. C shows that the first Lorentzian displays a peak broadening that shows a linear dependence with q^2, which indicates diffusion. D shows no q^2-dependence, which is indicative of localised constrained and restricted dynamics within the protein.

In accordance with the data analysis performed for $65.77\ \mu\text{eV}$ resolution, the reduced statistics of the incoherent data compared to the added data was again taken into account. For this purpose, the analysis of the incoherent data was repeated for an integration along the q-axis using $\Delta q = 0.15\ \text{Å}^{-1}$

as an increment. The final results for the added and the incoherent data are summarised in Table 1, with the diffusion coefficient as obtained from the slope of the peak broadening vs q[2] calculated for Equation 8 and Equation 10, while $\overline{\mathrm{HWHM}_1}$ was calculated for the peak broadening of Equation 8 and the peak broadening of the second Lorentzian of Equation 10 using the weighted mean. This value describes the quantitative value associated with the q^2-independent internal dynamics of the protein.

First, the roughly binned data was fitted with Equation 8, see Figure S 8. The data was described adequately $\chi^2 = 1.48$ by the fit and no $q^2 -$ dependence can be observed for the peak broadening.

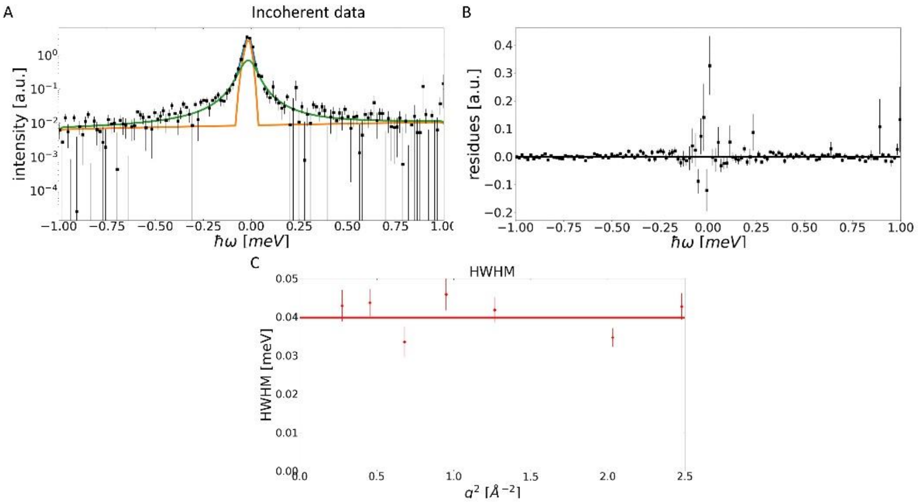

Figure S 8 A shows an example of the fit using Equation 8 for an incoherent spectrum at $q = 0.825$ Å^{-1}, for the resolution $30.8\ \mu eV$ using $\Delta q = 0.15$ Å^{-1}. The y-axis is logarithmic, the total fit function is shown in blue, the data as black dots, the Lorentzian in green and the elastic contribution in orange. All are convoluted with the instrument resolution. B shows the corresponding residues. C shows that the first Lorentzian displays a peak broadening that shows a linear dependence with q^2, which indicates diffusion. D shows no q^2-dependence, which is indicative of localised constrained and restricted dynamics within the protein.

To allow for a comparison to the total data the fit was repeated using Equation 10, see Figure S 9 $\chi^2 = 1.24$. Here the dangers of overfitting are again apparent as discussed above.

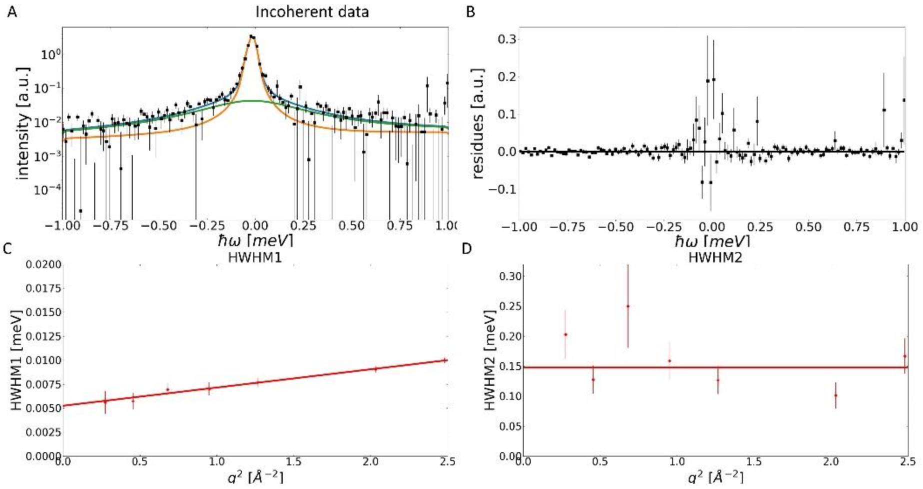

Figure S 9 A shows an example of the fit obtained using Equation 10 for an incoherent spectrum at $q = 0.825$ Å^{-1} for the resolution $30.8\ \mu eV$ using $\Delta q = 0.15$ Å^{-1}. The y-axis is logarithmic, the total fit function is shown in blue, the data as black dots, the Lorentzian in green and the elastic contribution in orange. All are convoluted with the instrument resolution. B

shows the corresponding residues. C shows the peak broadening for the incoherent data obtained using by plotting the spectra using Equation 10. Here it becomes apparent that no q^2-dependence is observed.

In addition, the HWM obtained from the repeated analysis of the total data for the limited q-range suggested in the main paper is displayed in Figure S 10. This shows impressively that no q^2-dependence exists for the total data when the suggested q-limit is applied. This means that the coherent contamination can be avoided.

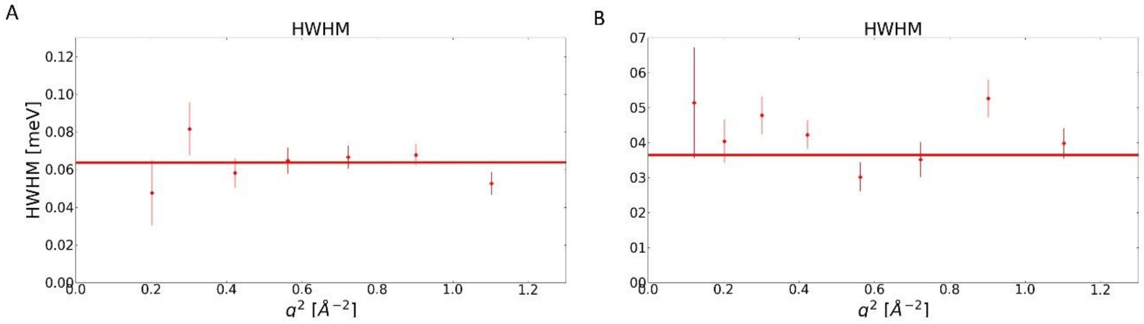

Figure S 10 (A) shows the now q^2-independent peak broadening of $65.77\ \mu eV$ resolution having applied the q-limits. This highlights that only the coherent contribution lead to the apparent q^2-dependent behaviour. (B) shows the now q^2-independent peak broadening of $30.8\ \mu eV$ resolution having applied the q-limits. This highlights that only the coherent contribution lead to the apparent q^2-dependent behaviour.

Bayesian analysis

In order to confirm that the chosen models are mathematically probable in addition to being justified on the grounds of the physical process expected to contribute to the dynamics, a Bayesian analysis using the Bayes analysis option in Mantid was performed[1]. Here, the posterior likelihood for the models corresponding to Equations 6 and 8 was calculated using the approach in[2]. The analysis was done for the coherent and incoherent data at both resolutions. To ensure that the difference in statistics between the total and the incoherent data does not affect the results the analysis was repeated for the incoherent data obtained for a wider q integration. This increases the statistics for each energy bin and should therefore compensate any difference in statistics between the total and incoherent data. It is further justified by the fact that any dynamics present are expected to be smooth

and slowly varying functions of q.

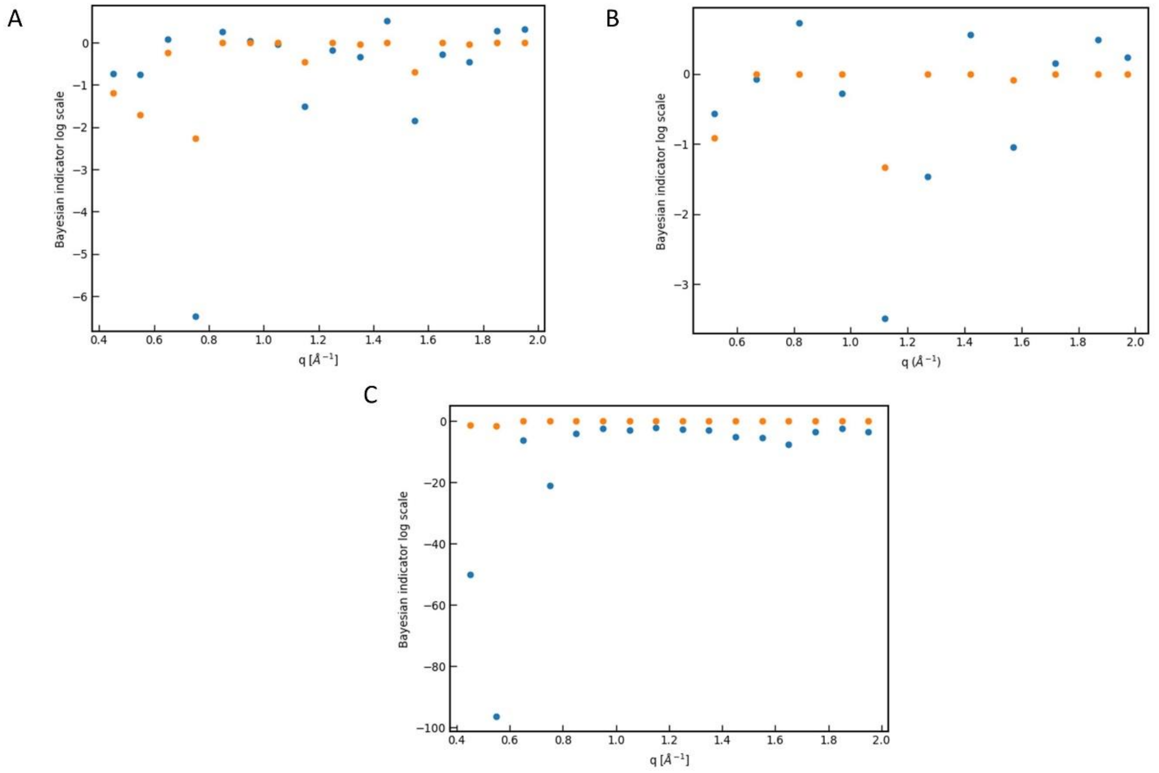

Figure S 11 All images show the Bayesian indicator for two Lorentzians (orange) or one Lorentzian (blue) according to Equation 8 and Equation 10 versus the associated q-values for the resolution of $65.77\ \mu eV$. The y-axis is given in log scale. (A) shows the results for the incoherent data with the normal q-integration range of $\Delta q = 0.1\ \text{Å}^{-1}$. (B) shows the results for the improved statistics incoherent data due to a q-integration range of $\Delta q = 0.15\ \text{Å}^{-1}$. (C) shows the results for the total data.

The Bayesian analysis confirms that for the incoherent data the one Lorentzian model given by Equation 8 (the single Lorentzian) is the most probable or equally probable for most q-values, as shown in Figure S *11* (A). Figure S *11* (B) indicates that Equation 8 becomes even more probable for improved statistics. On the other hand, the Bayesian analysis is not able to unambiguously distinguish between the models. The expected q^2-independent HWHM observed when fitting Equation 8 is now helpful in distinguishing between this and the model represented by Equation 10; the Bayesian prior probability for the former is considerably higher than the latter (although this is difficult to quantify). This leads us to the conclusion that Equation 8 remains the most appropriate choice for the incoherent data upon taking the Bayesian analysis into account.

For the total data as shown in Figure S *11* (C) the Bayesian analysis and the q^2-dependend HWHM upon trialling Equation 8 both indicate Equation 10 as the most appropriate choice. Clearly, this is because the presence of the coherent contribution. This shows that the Bayesian analysis confirms our choice of models and also that the reduced statistics for the incoherent data compared to the total data upon identical q-integration did not lead us to choose an inappropriate model. Adjusting the q-integration range for the incoherent data to achieve comparable statistics to the total data only strengthens the arguments for the choice of model.

A Bayesian analysis was repeated for the higher resolution of $30.8\ \mu eV$ as shown in Figure S *12* display the same behaviour and the same arguments apply. Thus the Bayesian analysis confirms the models chosen.

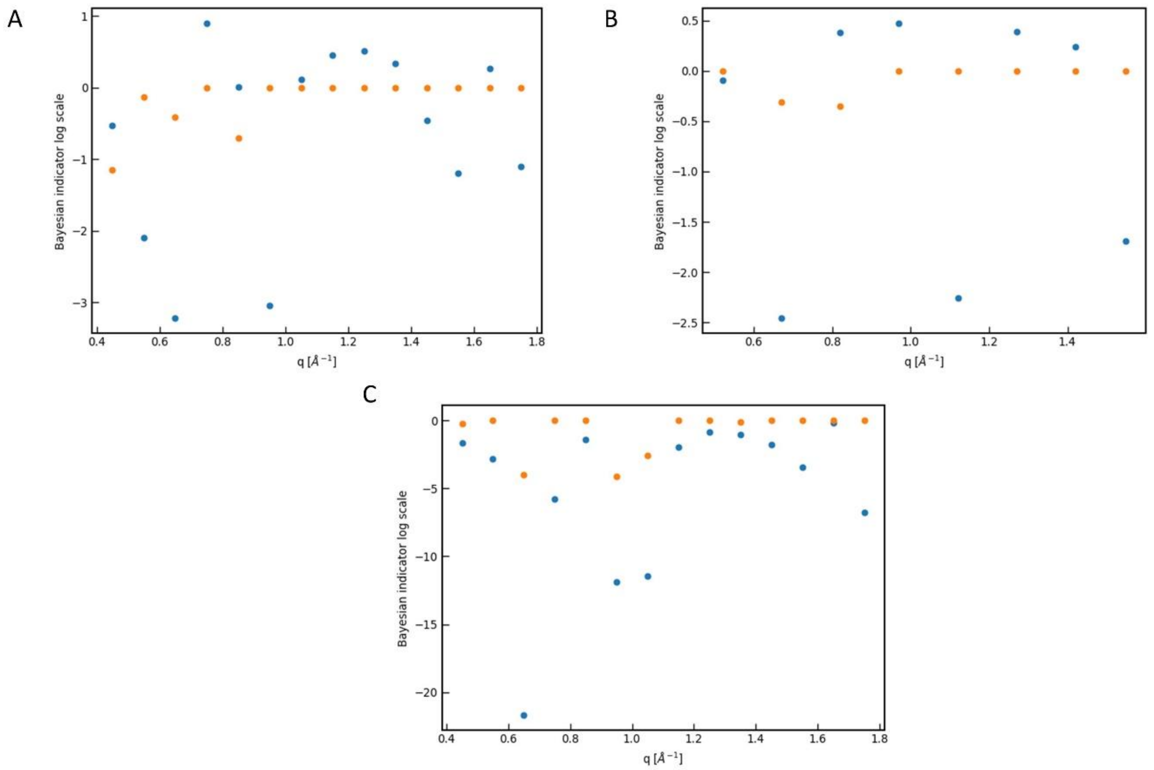

Figure S 12 All images show the Bayesian indicator for two Lorentzians (orange) or one Lorentzian (blue) according to Equation 8 and Equation 10 versus the associated q-values for the resolution of $30.5\ \mu eV$. The y-axis is given in log scale. (A) shows the results for the incoherent data with the normal q-integration range of $\Delta q = 0.1$ Å^{-1}. (B) shows the results for the improved statistics incoherent data due to a q-integration range of $\Delta q = 0.15$ Å^{-1}. (C) shows the results for the total data.

1. *Mantid (2013): Manipulation and Analysis Toolkit for Instrument Data*(2013)
2. DS Sivia, CJ Carlilie, WS Howells, S Koening *Bayesian analysis of quasielastic neutron scattering data*. Phys B 341–348(1992)
